# Differentiation and Functionality of Bone Marrow-Derived Mast Cells Depend on Varying Physiologic Oxygen Conditions

**DOI:** 10.3389/fimmu.2017.01665

**Published:** 2017-11-30

**Authors:** Helene Möllerherm, Karsten Meier, Kathrin Schmies, Herbert Fuhrmann, Hassan Y. Naim, Maren von Köckritz-Blickwede, Katja Branitzki-Heinemann

**Affiliations:** ^1^Department of Physiological Chemistry, University for Veterinary Medicine Hannover, Hanover, Germany; ^2^Faculty of Veterinary Medicine, Institute of Biochemistry, University of Leipzig, Leipzig, Germany; ^3^Research Center for Emerging Infections and Zoonoses (RIZ), University for Veterinary Medicine Hannover, Hanover, Germany

**Keywords:** mast cells, physioxia, HIF-1α, FOXO3, histamine, reactive oxygen species

## Abstract

Mast cells (MCs) are long-living multifunctional innate immune cells that originate from hematopoietic precursors and specifically differentiate in the destination tissue, e.g., skin, respiratory mucosa, intestine, where they mediate immune cell recruitment and antimicrobial defense. *In vivo* these tissues have characteristic physiological oxygen levels that are considerably lower than the atmospheric oxygen conditions (159 mmHg, 21% O_2_; 5% CO_2_) traditionally used to differentiate MCs and to study their functionality *in vitro*. Only little is known about the impact of physiological oxygen conditions on the differentiation process of MCs. This study aimed to characterize the differentiation of immature murine bone marrow-derived MCs under physioxia *in vitro* (7% O_2_; 53 mmHg; 5% CO_2_). Bone marrow-derived suspension cells were differentiated in the presence of interleukin-3 with continuous, non-invasive determination of the oxygen level using a Fibox4-PSt3 measurement system without technique-caused oxygen consumption. Trypan blue staining confirmed cellular viability during the specified period. Interestingly, MCs cultivated at 7% O_2_ showed a significantly delayed differentiation rate defined by CD117-positive cells, analyzed by flow cytometry, and reached >95% CD117 positive population at day 32 after isolation. Importantly, MCs differentiated under physioxia displayed a decreased transcript expression level of *hif-1*α and selected target genes *vegf, il-6*, and *tnf-*α, but an increase of *foxo3* and *vhl* expression compared to MCs cultivated under normoxia. Moreover, the production of reactive oxygen species as well as the amount of intracellular stored histamine was significantly lower in MCs differentiated under low oxygen levels, which might have consequences for their function such as immunomodulation of other immune cells. These results show for the first time that physioxia substantially affect maturation and the properties of MCs and highlight the need to study their function under physiologically relevant oxygen conditions.

## Introduction

Besides their well-known role in type I hypersensitivity reactions, mast cells (MCs) are multifunctional, long living sentinel cells with a key role in the innate immune response (1, 2). MCs derive from hematopoietic progenitor cells of the bone marrow (3), and circulate through the blood stream as immature precursors and lymphatics until they reach their final destination such as mucosal and epithelial tissues (4–6).

In the tissue, MC precursor differentiates under the influence of growth factors and cytokines that ultimately determine their mature phenotype (7). Two subtypes of MCs have been well characterized: mucosal MCs and connective tissue MCs (8). *In vitro* MCs differentiation is mediated by interleukin-3 (IL-3) leading phenotypically to the mucosal MC subtype (9). Murine bone marrow-derived MCs are commonly differentiated *in vitro* over 4–6 weeks by cultivating hematopoietic progenitor cells from murine bone-marrow in the presence of IL-3 (10). The differentiation status is generally confirmed by CD117/c-kit, a transmembrane protein with tyrosine kinase activity that regulates cell differentiation and proliferation (11). When 95% purity of mature MC suspension is reached, the cells are commonly used for functional studies (12–14). In the hematopoietic system, CD117/c-kit is expressed in most early progenitors including the stem-cell compartment (15). In most lineages, expression of CD117/c-kit is then lost during differentiation; only MCs maintain its expression throughout differentiation and maturation steps (16). For negative selection, Ly6G/C, small GPI-linked proteins on the surface of myeloid-derived cells, are used to identify neutrophils, monocytes, and granulocytes (17, 18).

*In vitro* experiments on the functionality of MCs are normally performed under normoxic oxygen levels with normoxic differentiated bone marrow-derived cells (oxygen conditions of *in vitro* cell culture systems: 150 mmHg or 19.95% oxygen, 20.3 kPa). Importantly, maturation of MCs occur *in vivo* under lower oxygen levels since the destination tissues of MCs, were the final differentiation takes place, vary in their physiological oxygen levels, depending on the local need of oxygen in the tissue (19): bone marrow (approx. 49 mmHg, 6.4%), blood (arterial blood: approx. 100 mmHg, 13.2%), intestinal mucosa and submucosa (approx. 57.6 mmHg 7.6%), alveolar wall and bronchi (gradient between 100 and 120 mmHg in the alveolus, 13.4–16%) (19).

Furthermore, during inflammatory processes, when MCs are involved, the physiological oxygen content of the tissue, termed physioxia, locally decreases to approx. 1% due to oxygen consumption by invading host immune cells and pathogens (20). This decreased physiological oxygen level in the tissue is called hypoxia (19). Interestingly, it has been shown that MCs functions significantly differ under acute hypoxia (1% oxygen) mimicking the oxygen content of acute inflamed tissue (21). MCs seem to rapidly adapt to low oxygen levels to better orchestrate the immune response under hypoxia by avoiding uncontrolled degranulation and tissue damage, e.g., by downregulating important proinflammatory cytokines like TNF-α and increasing the release of histamine (21). TNF-α and histamine are important proinflammatory effectors stored and released upon stimulation (22, 23). These mediators are immediately released by MCs in response to stimuli like bacteria including *S. aureus* to recruit other effector cells, i.e., neutrophils. Histamine increases the vascular permeability (24), while TNF-α acts as proinflammatory cytokine by recruiting and stimulating phagocytosis and degranulation of neutrophils (25). Since in those studies, the initial differentiation of MCs was performed under normoxic oxygen conditions, the question arises if MCs already display a distinct functional setup and gene expression when they differentiate *in vitro* in their physiological oxygen milieu.

To maintain homeostasis under low oxygen levels, the transcription factor HIF-1α is known to act as central regulator and key-player of the cellular adaptation to oxygen stress (26–28). HIF-1α activity is regulated by oxygen on the protein level controlling the transcription of numerous target genes: under normoxic conditions HIF-1α forms a complex with VHL for proteasomal degradation (29), whereas it is stabilized under low oxygen conditions and subsequently translocates into the nucleus to fulfill its regulatory functions (30). HIF-1α mediates inflammatory responses in various immune cells including MCs and also affects the bactericidal capacity (21, 30–34). Importantly, our own previous work revealed a minor role of HIF-1α in the short-term response of MCs to hypoxic 1% oxygen level (21). Therefore, it was of special interest to clarify the role of HIF-1α on MCs differentiation at physiological oxygen level.

We hypothesized that physiologic oxygen conditions affect the differentiation, cultivation, and functionality of MCs *in vitro*. Therefore, we compared the differentiation rate, transcript expression of *hif-1*α, selected target genes, *foxo3* and *vhl* as well as reactive oxygen species (ROS) production and histamine production as functional markers of MCs differentiated at 7% oxygen (here termed as physioxia) compared to MCs differentiated at atmospheric oxygen level (21%, here termed normoxia).

## Materials and Methods

### BMMC Isolation and Cultivation

Hematopoietic progenitor cells were isolated from femur and tibia of C57BL/6 wild-type (WT) mice. All progenitor cells derived from bones of one or two mice were pooled and again separated into two batches: one batch was cultivated under physioxic (7% O_2_; 53 mmHg; 5% CO_2_) and the other one under normoxic (21% O_2_; 159 mmHg, 5% CO_2_) conditions, respectively. Cells were differentiated into BMMCs (bone marrow-derived MCs) over 5–6 weeks in T25 suspension culture flasks (Sarstedt) under the influence of IL-3 (10 ng/ml) as previously described (35).

### BMMCs Differentiation Status and Viability

The purity and differentiation status of BMMCs was analyzed twice a week using flow cytometry (Attune NxT Flow Cytometer; ThermoFisher Scientific) by staining the MC marker CD117/c-kit using the antibody anti-mouse CD117, PE (phycoerythrin)-labeled (12 ng/10^5^ cells; Biolegend). Neutrophils, eosinophils, macrophages, and monocytes in the culture were stained by FITC conjugated rat anti mouse Ly-6G and Ly-6C antibody (25 ng/10^5^ cells; BD Pharmington), or its respective isotype controls. Cellular viability was determined twice a week by counting with a Neubauer counting chamber (Marienfeld) using trypan blue (Roth, 0.4% in PBS) discrimination. All functional assays were performed, if all BMMCs were tested as 95% CD117 positive (week 6/7 post-isolation).

### Oxygen Measurement

Oxygen was measured in 24-well plates (Nunc, Germany) using a Fibox4-PSt3 measurement system (PreSens Precision Sensing GmbH) as previously described (36). Oxygen measurements were performed over a time period of 48 h in cell culture media and on a weekly basis in the cell suspension during the 5-week differentiation process, while the cells were incubated under physioxic (7% O_2_; 53 mmHg; 5% CO_2_) or normoxic (21% O_2_; 159 mmHg, 5% CO_2_) conditions, respectively.

### Histamine Determination in the Cellular Pellet

Histamine levels in the cellular pellets of physioxic and normoxic differentiated BMMCs were calculated for a total of 1 × 10^6^ BMMCs. Cells were incubated in equilibrated complete HBSS (containing 25 mM HEPES and 0.1% BSA) under physioxic (7% O_2_; 53 mmHg; 5% CO_2_) or normoxic (21% O_2_; 159 mmHg, 5% CO_2_) conditions, respectively. BMMCs were treated and prepared as described earlier (21) with the MC-degranulating peptide mastoparan (50 µM; Bachem, Heidelberg, Germany) as a positive control or without external stimulation (spontaneous release) for 45 min in a volume of 100 µl complete HBSS. The histamine content was measured by rpHPLC as previously described by Gueck et al. (37).

### RNA Expression Analysis

Previous to RNA isolation 1 × 10^6^ BMMCs were incubated under normoxia or physioxia for 3 h in IMDM (supplemented with 0.1 mM MEM and 2% of 70°C h.i. FCS) in a 1.5-ml reaction tube. RNA was extracted with the RNeasy Mini Kit (Qiagen) by following the user’s manual. The quality of RNA was tested with a bioanalyzer (RNA 6000 Pico Kit, Agilent) as described in the manufacturer’s instructions. The RNA quality scoring and RT-qPCR was done as previously described (21). Finally, physioxia and normoxia values were normalized to the housekeeping gene *rps9* using the ΔCt method: Ct (cycle threshold) is defined as the number of cycles required for the fluorescent signal to cross the threshold. Ct levels are inversely proportional to the amount of target nucleic acid in the sample (i.e., the higher the Ct level the lower the amount of target nucleic acid in the sample). Respective oligonucleotide primers used for RT-qPCR are listed in Table 1.

**Table 1:** Oligonucleotide primers used in RT-qPCR.

Primer	Accession number	Sequence (sense, antisense)	RNA/DNA	Tm°C
*rsp9*	NM_029767	Forward primer TTGTCGCAAAACCTATGTGACC	147/344	61.1
		Reverse primer GCCGCCTTACGGATCTTGG		62.8

*tnf-α*	X02611	Forward primer CCTGTAGCCCACGTCGTAG	148/442	61.5
		Reverse primer GGGAGTAGACAAGGTACAACCC		61.4

*il-6*	NM_031168	Forward primer CTGCAAGAGACTTCCATCCAG	131/–	60.1
		Reverse primer AGTGGTATAGACAGGTCTGTTGG		60.8

*hif-1*α	NM_001313919.1	Forward primer CATCCAGAAGTTTTCTCACACG	138/–	63.5
		Reverse primer GGCGAAGCAAAGAGTCTGAA		64.5

vegf	AY707864.1	Forward primer AGTCCCATGAAGTGATCAAGTTCA	220/730	65.7
		Reverse primer ATCCGCATGATCTGCATGG		67.2

*foxo3*	NM_019740.2	Forward primer CTGCTCGTGGAAGGGAGGAGGA	181/–	65.43
		Reverse primer GAGCTCCAGCTCGGCTCCTT		64.10

*vhl*	NM_009507.3	Forward primer GCGAATCCGAGGGACCCGTT	435/–	64.69
		Reverse primer TGACCAGGCTCCGCACAACC		65.15

### ROS Determination

A total of 2 × 10^5^ BMMCs were centrifuged at 90 × *g* and resuspended in 500 µl oxygen equilibrated IMDM (supplemented with 0.1 mM MEM and 2% of 70°C h.i. FCS) as negative control, phorbol 12-myristate 13-acetate (PMA, 25 nM; Sigma) in IMDM as positive control. BMMCs, differentiated under physioxia (159 mmHg, 21% O_2_; 5% CO_2_) were stimulated for 45 min in a 1.5-ml reaction tube. BMMCs, differentiated under normoxia (7 mmHg, 1% O_2_; 5% CO_2_) were stimulated for 45 min in normoxic oxygen levels, respectively. For further examinations, normoxically differentiated cells were preincubated 3 h under physioxia or normoxia before stimulation. ROS production was quantified by dichlorofluorescein (DCF; Sigma) using flow cytometry (Attune NxT Flow Cytometer; ThermoFisher Scientific). DCF was added at a final concentration of 10 µM, 15 min before the treatment incubation ended, for a total of 30 min. Mean green fluorescence intensity of all (X-Mean of BL-1) was recorded and represents the mean ROS production.

### Statistical Analysis

If not indicated otherwise for each experimental setup, at least three independent experiments using at least three MC batches were performed. Data analysis was performed using Excel 2010 (Microsoft) and GraphPad Prism 7.0 (GraphPad Software). Differences between two groups were analyzed by using an unpaired, two-tailed Student’s *t*-test, if not otherwise stated. The significance is indicated as follows: ns, not significant, *p ≤ 0.05, **p ≤ 0.01, ***p ≤ 0.001, and ****p < 0.0001.

## Results

### Oxygen Concentration in the Cell Media and Culture Suspension

**Figure 1:**
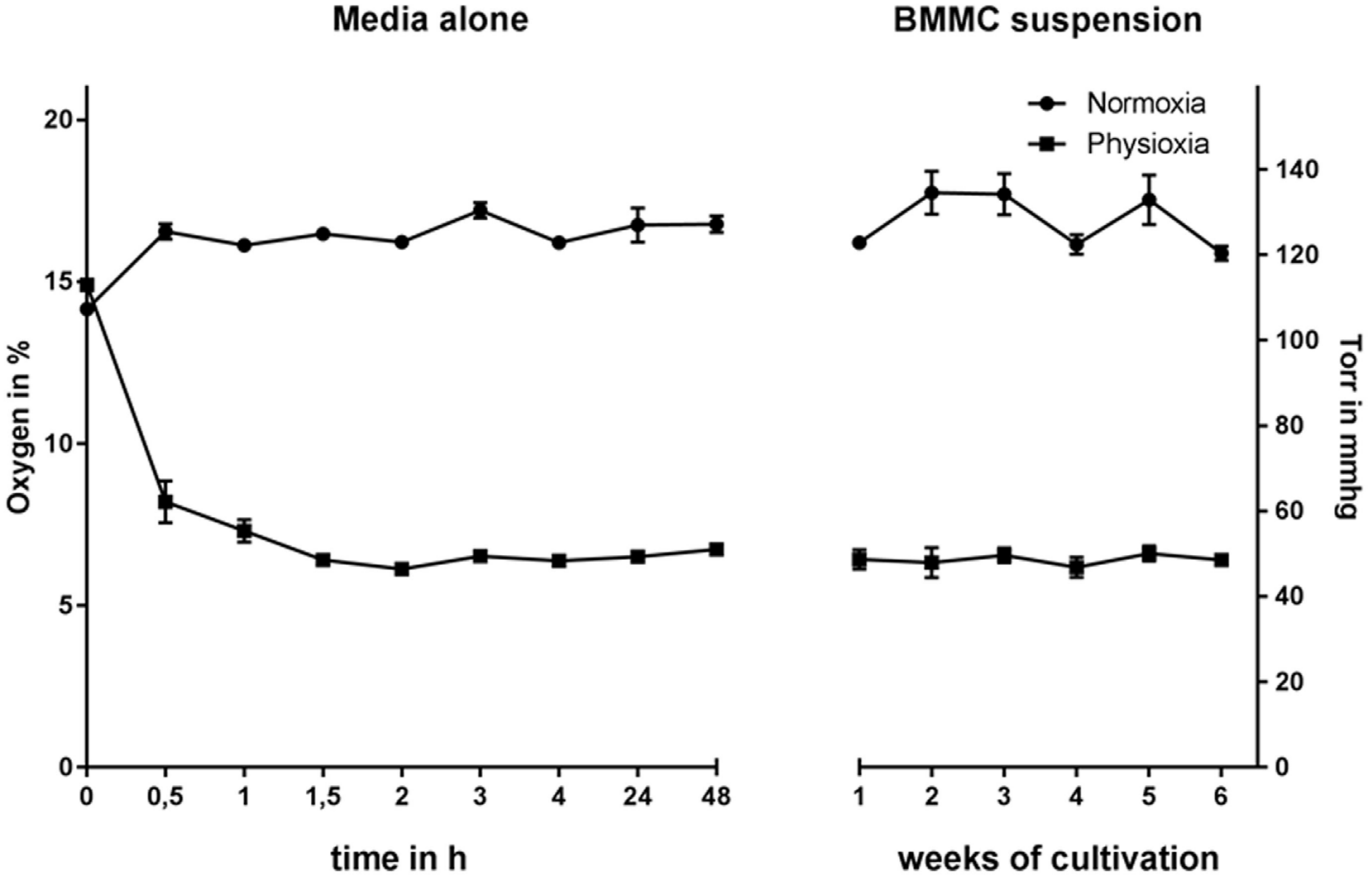
Oxygen concentration in media without (left, monitored up to 48 h under physioxia) and in BMMC suspension culture (right, monitored every week) under normoxia and physioxia, measured using a Fibox4-PSt3 measurement system (PreSens^®^). The oxygen level of both media alone as well as in the cell suspension stabilizes at around 53 mmHg (7% O_2_) und physioxia, while the normoxic remains above 120 mmHg (16% O_2_). Equilibration of the media is reached after approx. 1.5 h. Depicted are the mean values and SDs of *n* = 4 experiments with cells derived from 4 mice (not pooled).

Hematopoietic progenitor cells were isolated from the bone marrow of C57BL/6 WT mice and differentiated in the presence of IL-3 for 39 days under physiological oxygen conditions in comparison to atmospheric oxygen conditions. The experimental settings of cultivating MCs under physioxia were monitored over the whole differentiation period by measuring the oxygen content in the cell suspension with continuous, non-invasive determination of the oxygen level using a Fibox4-PSt3 measurement system (PreSens^®^). The oxygen level stabilizes at around 53 mmHg (7% O_2_), while the normoxic remains above 120 mmHg (16% O_2_) in media alone as well as in the cell suspension (Figure 1), confirming that MCs do not massively consume oxygen in the culture media during differentiation. To evaluate the time needed for the media to equilibrate to physioxic or normoxic oxygen levels, the oxygen content was monitored up to 48 h under hypoxia. The oxygen level equilibrates after 1.5 h and stays constant for up to 48 h (Figure 1).

### Cell Viability

**Figure 2:**
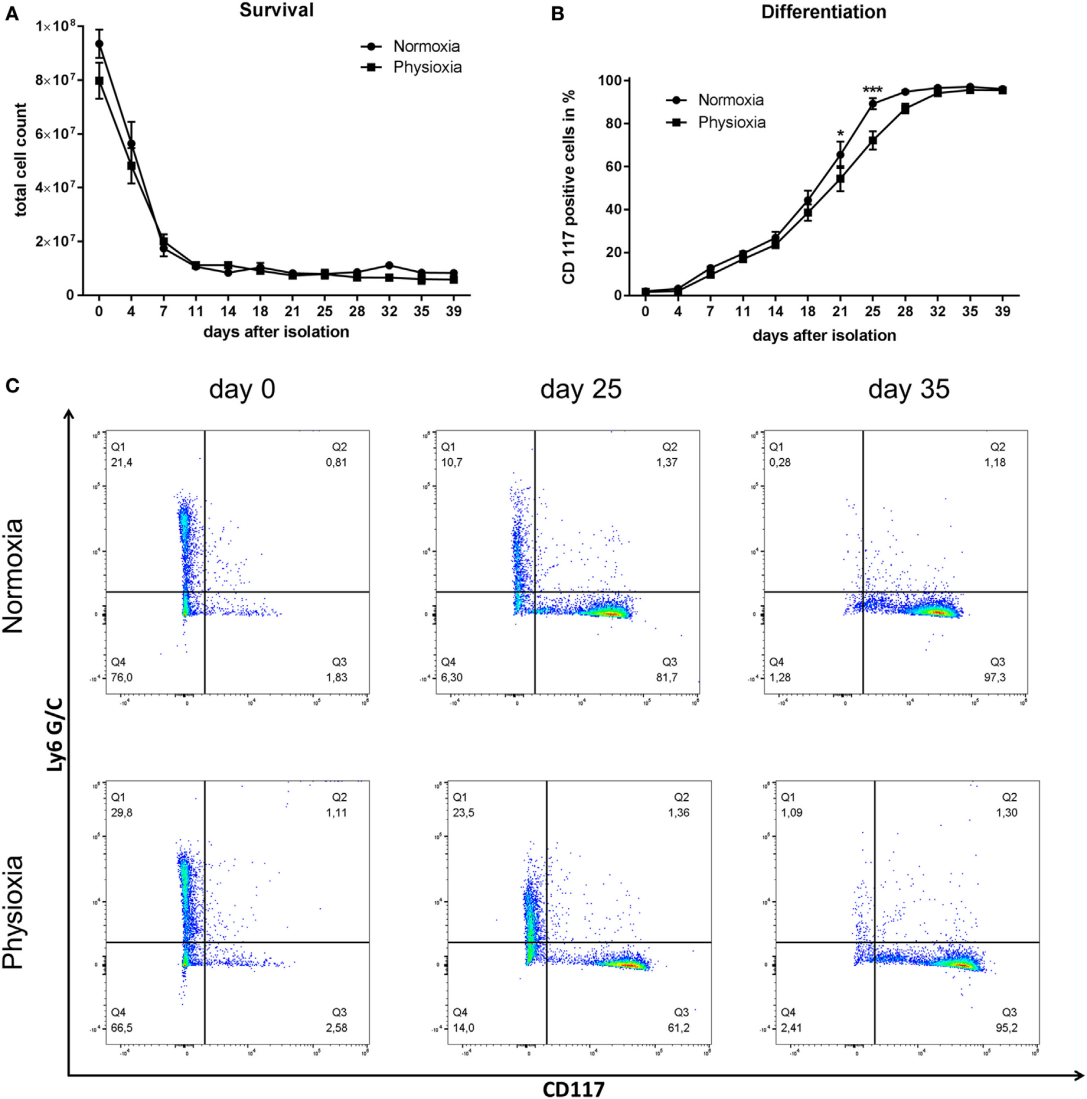
**(A)** Total cell count of viable cells was determined by counting with a Neubauer counting chamber using trypan blue discrimination. No significant differences in cell survival and cell number are observable under physioxia compared to normoxia. The initial loss of cells in the first week of cultivation is due to strong selection of the primarily very diverse bone marrow-derived cell population. Depicted are mean values and SEM from cell suspensions of *n* = 3 separated BMMC-batches derived from three mice (not pooled). **(B)** Time-dependent CD117 expression as marker for mast cell (MC) differentiation. Increasing CD117 expression over time indicates progressing MC differentiation that is slightly but significantly delayed under physioxic culture conditions compared to normoxia at day 21 and 25 post-isolation. Depicted are mean values and SEM from cell suspensions of *n* = 3 separated BMMC-batches derived from three mice (not pooled). **(C)** Representative dot blot diagrams of Ly6 G/C- and CD117-labeled cells on day 0, 25, and 35 post-isolation, cultivated under normoxia or physioxia. Cells were stained for the surface markers Ly6 G/C and CD117. Q1: Ly6 G/C positive cells, Q2: double positive cells, Q3: CD117 positive cells, Q4: double negative cells. Independent of the oxygen level, the number of Ly6 G/C-positive cells decreases while the amount of CD117-positive cells increases during cultivation.

The viability of MC progenitor cells under physioxia compared to normoxia was evaluated by counting the cells in trypan blue. Total cell number decreases in the beginning and remains constant at approx. 1 × 10^7^ 10 days post-isolation under normoxia and physioxia, respectively (Figure 2A). The primary loss of cells in the first week of cultivation is due to strong selection of the initially very heterogeneous bone-marrow derived cell population. After this initial loss of mainly adherent and immature immune cells that might die due to a lack in, e.g., growth factors, the cell number stays constant, indicating that no significant differences in cell survival and cell number are observable under physioxia compared to normoxia.

### Differentiation Status of MCs

The differentiation status was monitored using flow cytometry with cell-specific surface markers: Ly6 G/C is a marker for neutrophils and monocytes and CD117/c-kit receptor for mature MCs. Increasing CD117 expression over time indicates progressive MC differentiation that is slightly but significantly delayed under physioxic culture conditions compared to normoxia at day 21 (mean difference to normoxic differentiated cells: 11.1%) and 25 (mean difference to normoxic differentiated cells: 17.08%) post-isolation. Final differentiation rate averages more than 95% after 39 days in all samples (Figures 2B,C). Independent of the oxygen level, the number of Ly6 G/C-positive cells decreases, while the amount of CD117-positive cells increases during cultivation (Figure 2C).

### Transcription Expression of Selected Target Genes Known to Modulate Cellular Function in Response to Oxygen

**Figure 3:**
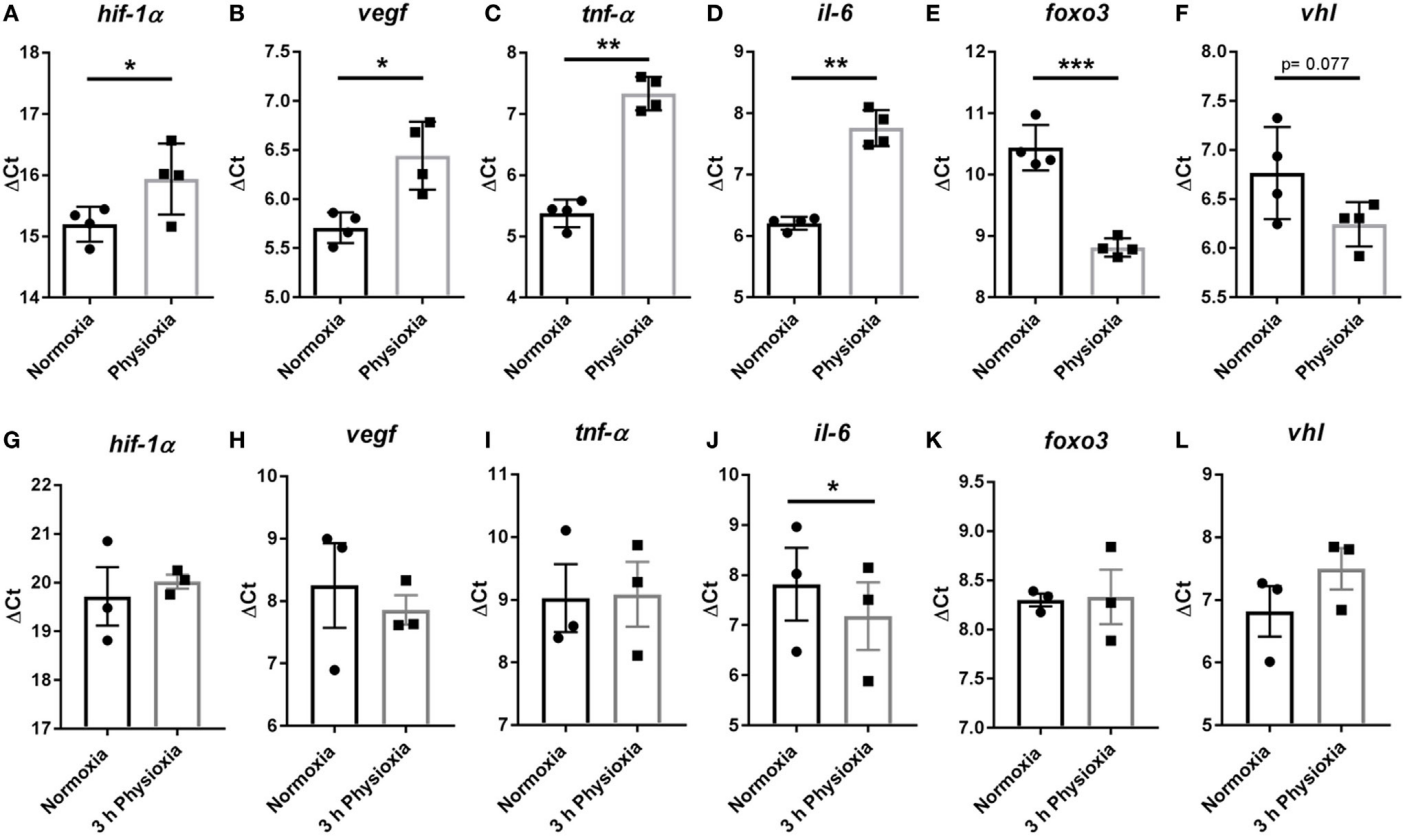
**(A–D)** Transcript expression of *hif-1*α, *vegf, tnf-*α, and *il-6* in MCs differentiated under physioxia compared to normoxia. MCs differentiated under physioxia display a significant lower transcript expression of *hif-1*α, *tnf-*α, *il-6*, and *vegf* compared to normoxia. Transcript expression of **(E)** *foxo3* and **(F)** *vhl* in MCs demonstrate that *foxo3* is significantly upregulated by comparing physioxic versus normoxic differentiated MCs. The expression of *vhl* shows a tendency (*p* = 0.077) to be upregulated under physioxia compared to normoxia. Depicted are the mean values and SDs of *n* = 4 experiments with cells derived from four mice (not pooled). **(G–L)** Transcript expression of *hif-1*α, *vegf, tnf-*α, *il-6, foxo3*, and *vhl* in MCs of normoxic differentiated cells and a following short-term (3 h) stimulation under physioxia compared to normoxia. No difference in ΔCt values of *hif-1*α, *tnf-*α, *vegf, foxo3*, and *vhl* was observable. Expression of *il-6* was significantly increased under physioxia compared to normoxia. Physioxia and normoxia values were normalized to the housekeeping gene *rps9* using the ΔCt method: Ct (cycle threshold) is defined as the number of cycles required for the fluorescent signal to cross the threshold. Ct levels are inversely proportional to the amount of target nucleic acid in the sample (i.e., the higher the Ct level the lower the amount of target nucleic acid in the sample). Depicted are the mean values and SDs of *n* = 3 experiments with cells derived from six mice (two mice pooled for each batch). Statistical differences were analyzed using a paired, two-tailed Student’s *t*-test.

To evaluate possible changes in gene expression of differentiated mature MCs, the transcript expression of *hif-1*α and some selected regulated HIF-1α target genes were measured *via* qRT-PCR at day 39 after isolation when cells were more than 95% positive for CD117. MCs differentiated under physioxia exhibit a significantly lower transcript expression of *hif-1*α (Normoxia ΔCt 15.2 ± 0.14 versus physioxia ΔCt 15.94 ± 0.29), *tnf-*α (Normoxia ΔCt 5.38 ± 0.11 versus physioxia ΔCt 7.33 ± 0.14), *il-6* (Normoxia ΔCt 6.20 ± 0.053 versus physioxia ΔCt 7.75 ± 0.15), and *vegf* (Normoxia ΔCt 5.71 ± 0.08 versus physioxia ΔCt 6.44 ± 0.17) compared to normoxia, depicted here as ΔCT values (Figures 3A–D).

Besides HIF-1α, the FOXO3 transcription factor is known to play a role in adaptation to oxygen stress (38, 39). To unravel if FOXO3 is differentially expressed during physioxic differentiation in MCs and if it may have an effect on HIF-1α degradation through the direct upregulation of *vhl*, gene expression of *foxo3* and its recently shown target *vhl* was conducted (39). As shown in Figure 3E, the expression of *foxo3* was significantly upregulated comparing physioxic (ΔCt 8.81 ± 0.075, *n* = 4) versus normoxic (ΔCt 10.44 ± 0.18, *n* = 4) differentiated MCs. The expression of *vhl* shows a tendency (*p* = 0.077) to be downregulated under physioxia (ΔCt 6.24 ± 0.11, *n* = 4) compared to normoxia (ΔCt 0.77 ± 0.23, *n* = 4) (Figure 3F).

To confirm that the difference in gene expression is a direct effect of differentiation under physioxia, short-term physioxia was used for verification of gene expression of *hif-1*α, *tnf-*α, *il-6, vegf, foxo3*, and *vhl* compared to normoxia. The transcript expression of MCs differentiated under normoxia and challenged for 3 h with 7% O_2_ was evaluated. No difference in ΔCt in *hif-1*α, *tnf-*α, *vegf, foxo3*, and *vhl* was observed (Figures 3G–I,K,L). Even so, the expression of *il-6* was significantly increased after short-term incubation under physioxia (normoxia: ΔCt 7.82 ± 0.73, *n* = 3; physioxia: ΔCt 7.18 ± 0.67, *n* = 3) (Figure 3J).

### Generation of ROS

**Figure 4:**
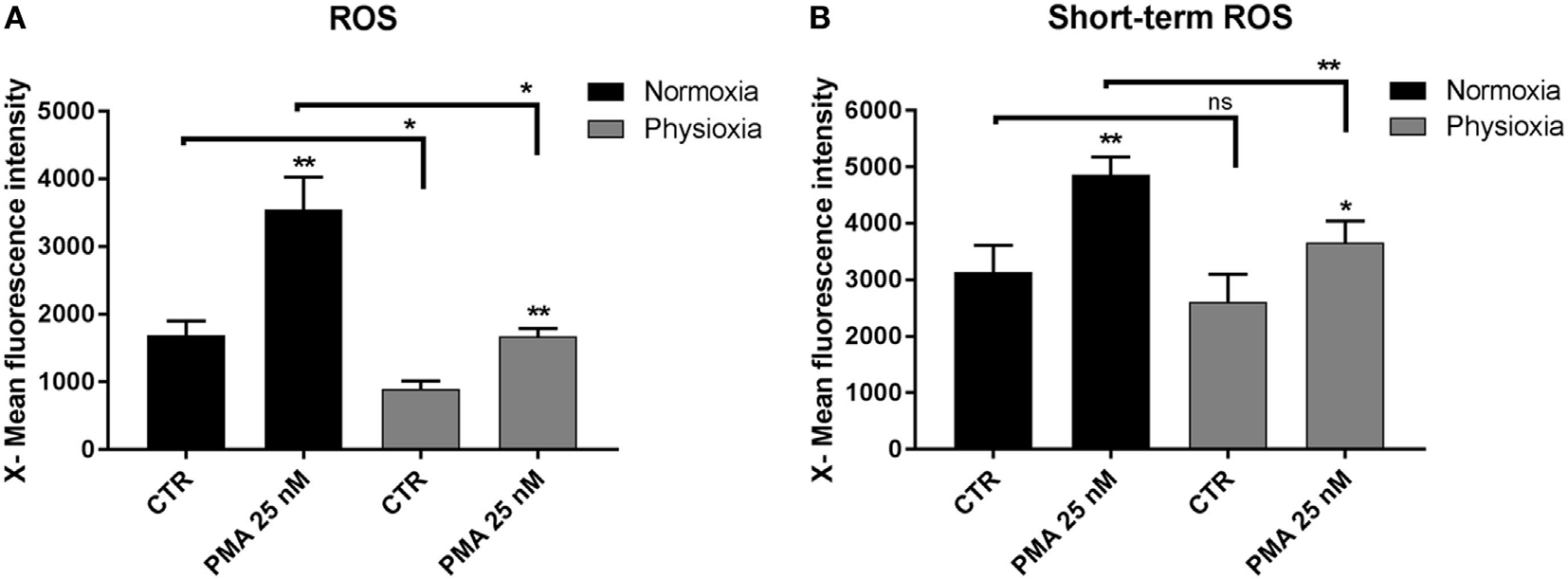
**(A)** Reactive oxygen species (ROS) production of mast cells (MCs) differentiated under physioxia compared to normoxia. The results display that PMA (25 nM) induces ROS production in MCs in both conditions. By comparing physioxic versus normoxic differentiated MC, ROS production is significantly and drastically reduced. Depicted are mean values and SEM from cells of *n* = 4 (eight mice, two mice pooled for each batch) experiments for normoxic cultivated MCs and *n* = 3 (six mice, two mice pooled for each batch) for physioxic cultivated MCs. **(B)** ROS production of MCs differentiated under normoxia and pre-incubated for 3 h (short-term) under physioxia in comparison to normoxia. PMA stimulated MCs significantly produce more ROS under short-term physioxia and normoxia. By comparing short-term physioxia versus normoxia, PMA stimulated ROS production is significantly decreased under physioxia. Depicted are mean values and SEM from cells of *n* = 4 (eight mice, two mice pooled for each batch) experiments. ROS production was analyzed by flow cytometry using the DCF-fluorescence based method. Depicted is the X-mean green fluorescence intensity of each cell. Differences within one group were analyzed by using a paired, two-tailed Student’s *t*-test.

Reactive oxygen species are essential mediators of inflammation, playing a role in innate immune signaling (40) and have a direct biocidal effect on invading bacteria. Detrimental effects are also known: ROS may damage the host cells (41). Therefore, ROS production must be tightly regulated for maintaining host cell homeostasis in case of infection. ROS production in MCs differentiated under physioxia or normoxia was analyzed by flow cytometry by conducting the X-Mean green fluorescence intensity of each cell (Figure 4A). The results show that PMA (normoxia: 3,553 ± 476.1, *n* = 4; physioxia: 1,674 ± 116.7, *n* = 3) significantly induces ROS production under both conditions in comparison to the unstimulated control (normoxia: 1,691 ± 210, *n* = 4; physioxia: 899 ± 114, *n* = 3). By comparing physioxic versus normoxic differentiated MC, overall ROS production was significantly reduced when cells were differentiated at 7% oxygen in the control group, this effect was not significant when cells were incubated only for 3 h at 7% oxygen.

To test, whether this effect is due to differentiation of MCs under physioxia or due to physioxic oxygen alone, short-term ROS production was measured with normoxically differentiated MCs after 3 h exposure to physioxia (Figure 4B). In this experiment, also PMA stimulated MCs significantly to produce ROS under short-term physioxia and normoxia (normoxia: 4,859 ± 316.3, *n* = 4; physioxia: 3,658 ± 382.3, *n* = 4) in comparison to their respective control (normoxia: 3,138 ± 475.1, *n* = 4; physioxia: 2,611 ± 486, *n* = 4). By comparing short-term physioxia versus normoxia, PMA stimulated ROS production is significantly decreased under physioxia. Overall a decreased ROS production was more pronounced in MCs differentiated under physioxia, thus confirming the direct effect of physioxic differentiation on ROS production.

### Intracellular Histamine Levels

**Figure 5:**
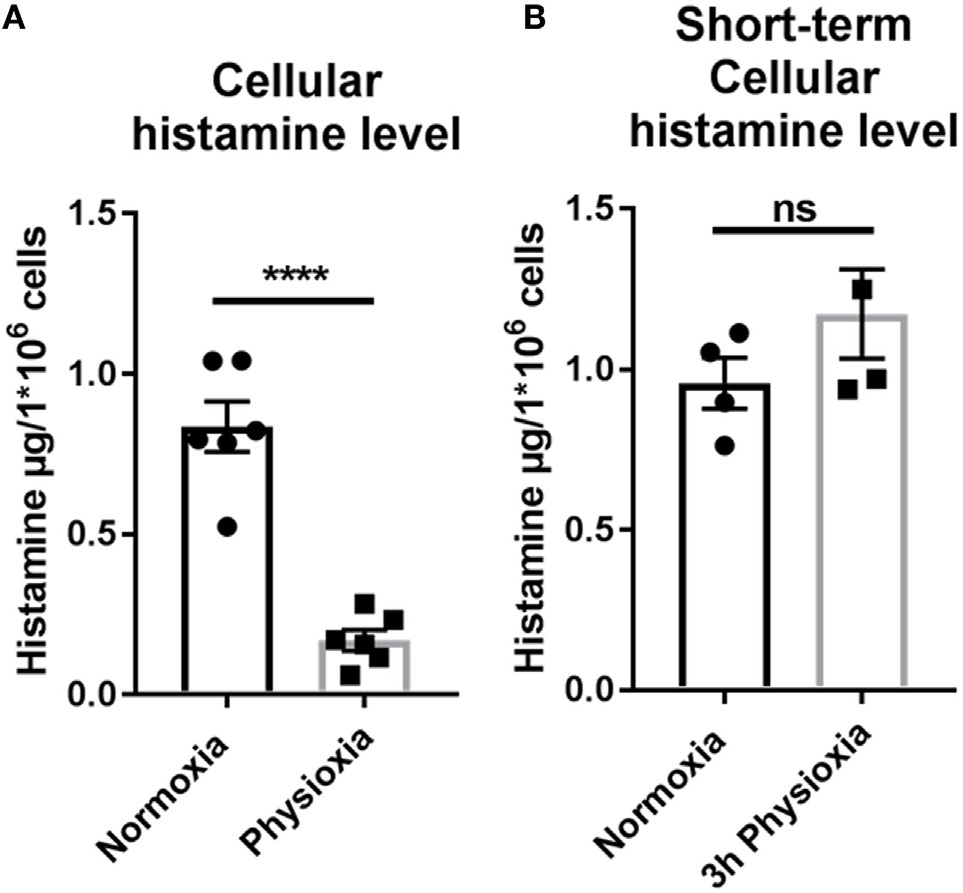
**(A)** Intracellular histamine storage after physioxic differentiation measured by high performance liquid chromatography. The amount of intracellular stored histamine is significantly reduced in MCs differentiated under low oxygen levels. Depicted are mean values and SEM from cells of *n* = 6 individual BMMC batches. **(B)** Intracellular histamine storage of normoxic differentiated cell after short-term (3 h) physioxia in comparison to normoxia. No difference in intracellular storage of histamine is observed. Depicted are single values and SEM from cells of minimum *n* = 3 (six mice, two mice pooled for each batch) individual experiments.

Histamine is a key mediator in effector cell recruitment and is released by MCs. The impact of physioxia on the functionality of MCs after maturation under physiologic oxygen conditions was ascertained by investigating histamine levels with high performance liquid chromatography (HPLC). The amount of intracellular histamine is significantly reduced in MCs differentiated under low oxygen levels (Figure 5A). Under normoxia, a mean of 0.89 ± 0.078 µg histamine/ml was measured, while under physioxia this level is reduced (0.12 ± 0.03 µg histamine/ml). To determine if this effect is specific for physioxic differentiation, cellular histamine level after short-term physioxia (3 h) was measured in normoxically differentiated cells (Figure 5B). No difference in histamine storage could be observed (normoxia: 0.96 ± 0.079, *n* = 4; physioxia: 1.173 ± 0.140, *n* = 4), confirming the effect of physioxic differentiation.

## Discussion

It is well known that reduced oxygen levels, which predominantly occur during infection and inflammation, have an important impact on immune cell functions *in vitro* (21, 30–34). However, the importance of oxygen on differentiation of MCs *in vitro* is still not completely understood, although it is well known that the physiological oxygen level in resident tissues—the place where immature precursors differentiate to mature MCs—are much lower than atmospheric oxygen concentrations (19). Nowadays, the importance of this fact is increasing, especially regarding the reproducibility of *in vitro* culture systems when focusing on oxygen levels in immunological studies, as recently reviewed by Zenewicz (42). Our own previous studies with neutrophils or MCs revealed that there is an immune cell-specific response to hypoxic (1%) oxygen conditions that differs from the normoxic phenotype (21, 34). Here, we investigated the impact of reduced physioxic (7%) oxygen level on the differentiation of bone marrow-derived hematopoietic stem cells into mature MCs.

By cultivating MCs under physioxia (7% O_2_), no difference in cell viability and cell number was observed over 39 days of differentiation compared to normoxic (21% O_2_) differentiation (Figure 2A). Interestingly, for other cell types, it is known that physioxia and prolonged hypoxia influence the cellular survival: e.g., human fibroblasts increase their lifespan by 25% under 10% O_2_ compared to normoxically cultivated cells (43). For primary neutrophils, it was also shown that culturing under hypoxia protects from apoptosis (33, 44). In stem cell biology, the culturing of cells under low oxygen already found its way into routine. Culturing embryonic stem cells at 5% oxygen retains their pluripotency (45). Importantly, Gornostaeva and colleagues conclude from their results that “the ‘hypoxic’ phenotype of ASCs (allogeneic adipose tissue-derived multipotent mesenchymal stem cells) may be more ‘desirable’ for the interaction with allogeneic immune cells” (46). In addition, oxygen is considered as an important signaling molecule, which has an integral role in the hematopoietic stem cell maintenance as well as in stem cell proliferation and differentiation (47).

Although no difference in cell viability was observed in this study (Figure 2A), the differentiation rate was found to be significantly slower on day 21 and 25 post-isolation under physioxic compared to normoxic differentiated MCs (Figures 2B,C). Interestingly, Köhler et al. (48) showed in 2012 that the differentiation of bone marrow-derived dendritic cells (BMDCs) under hypoxia (1% O_2_) leads to enhanced phenotypic maturation into mature BMDCs but reduced cell growth and production of several cytokines. This was independent of HIF-1α, as shown by using mice with a conditional deletion of HIF-1α in dendritic cells. In good correlation to these data, we found that the transcript expression of *hif-1*α and selected target genes is downregulated in MCs cultured under physioxia (Figures 3A–D). The data support the assumption that under physioxia HIF-1α plays only a minor role during MC differentiation.

Possible candidates for an alternative response to stress stimuli including oxygen stress are Forkhead transcription factors (FOXO) (38). Mammalian cells express four FOXO factors: FOXO1, FOXO3a, FOXO4, and FOXO6. These factors are evolutionarily conserved and also identified in *C. elegans*, here named as DAF-16. DAF-16 promotes a developmental stage of *C. elegans* that ensures survival under nutrient-poor conditions (49). FOXO3 is the main factor detected in the thymus and bone marrow (38). Beside their role as tumor suppressors in several systems (50), they are identified to play a role in the adaptation of the cell to hypoxic stress. Thereby, especially FOXO3 antagonizes cMyc function, which results in downregulation of mitochondrial activity, thus preventing hypoxia-induced ROS formation which would consequently result in HIF-1α stabilization (51–53). We show here, that *foxo3* expression is significantly increased in MCs cultivated under physioxia (Figure 3E), along with reduced *hif-1*α expression (Figure 3A), suggesting that FOXO3 plays also a role in differentiation of MCs under physioxia and may also play a role in the downregulation of *hif-1*α. Controversially to this suggestion, it was published that FOXO3 is activated downstream of HIF-1α in fibroblasts as well as in breast cancer cells (54). Consequently, MCs upregulation of *foxo3* might occur in a HIF-1α-independent manner or even HIF-1α degradation occurs. Nevertheless, further studies are needed to unravel the role of FOXO3 and HIF-1α as well as their interplay during physioxic differentiation of innate immune cells.

Liu et al. recently showed in a zebrafish model that FOXO3 directly binds to VHL, an E3 ubiquitin ligase complex that is responsible for HIF-1α degradation. They stated that in zebrafish the disruption of *foxo3b* (the homolog of *foxo3* in human) led to impaired hypoxic tolerance and discussed that this might also be due to the influence of *foxo3b* on HIF activity *via* regulation of *vhl*. The expression of *vhl* is downregulated in MCs differentiated under physioxia, though this effect is not significant (Figure 3F). Possibly, MCs differentiate under physioxia by expressing *foxo3* and *vhl*, leading to degradation of HIF-1α.

Besides antimicrobial activities of immune cells, like phagocytosis, mediator-, and extracellular traps release, ROS play a central role in directly killing pathogens or mediating inflammation (55). ROS include, e.g., superoxide anions $\left({\text{O}}_{2}^{\cdot -}\right)$, hydrogen peroxides (H_2_O_2_), and hydroxyl radicals (OH^⋅^), and others that confer reactivity to different biological targets (56). One example is lysosomal digestion of pathogens after phagocytosis, by enzymes and *H_2_O_2_* (57). Moreover, ROS stimulate the production of proinflammatory cytokines (58) and are discussed as second messenger in signal transduction (59, 60). Increased production results in hyperactivation of inflammatory responses, tissue damage and disease (41). MCs are well known to be strongly involved in acute lung injury and tissue damage due to MC degranulation and systemically circulating ROS (61). The additional production of ROS by activated MCs tremendously alters the risk for injury severity; therefore, ROS-generation needs to be strictly controlled. MCs differentiated under physioxia produce significantly less ROS without or with stimulus (PMA) (Figure 4A). Since it is published that acute hypoxia increases ROS production in MCs and various other cell types (62), ROS production might be otherwise regulated and strictly controlled in MCs during physioxic differentiation. A possible explanation might be the relation of mitochondrial ROS production and FOXO3. As already mentioned, FOXO3a could be a down-regulator of HIF-1α. Interestingly, FOXO3a activation blocks the hypoxia-dependent increase in ROS in colon cancer cells and prevents HIF-1α stabilization (53). In another study, it was demonstrated that FOXO3a protects quiescent cells from oxidative stress by inducing manganese superoxide dismutase (Mn-SOD), leading to less ROS. The fact that hypoxia increases cellular ROS production, could lead to the conclusion that the decreased ROS level under physioxia could prevent detrimental accumulation of ROS in MCs. One hind for this hypothesis is the increased expression level of *foxo3* during physioxic differentiation, which could decrease ROS by inducing the expression of antioxidant enzymes, like Mn-SOD. By analyzing ROS production in response to short-term physioxia, we could also confirm that this effect is due to physioxic differentiation.

A key function of MCs is the release of mediators to mediate the recruitment of effector cells and orchestrate the immune response. One of the most important mediators of MCs is histamine, which was shown to be significantly and drastically decreased intracellularly under physioxia, suggesting an impact of physioxic differentiation on secretory granule composition. Histamine increases vascular permeability to enhance the blood flow and to allow the recruitment of other effector cells. Furthermore, histamine enhances epithelial cell mucus production to avoid bacterial residence (1). The observed decreased histamine storage after differentiation under physioxia (Figure 5A) may decrease the vasoactive capability of MCs, thus antimicrobial activity during infection is impaired. The intracellular storage after short-term physioxic incubation was not affected, thus the effect of physioxic differentiation on histamine storage was confirmed (Figure 5B). Nevertheless, histamine storage was reduced after physioxic differentiation. However, the histamine release in response to mastoparan was still observable, thus MCs are still responsive (Figure S1 in Supplementary Material). It was shown in addition that gene expressions of important pro-inflammatory cytokines, i.e., *il-6* and *tnf-*α and also *vegf*, one of the most important vascular permeability factors, were downregulated after physioxic differentiation (Figures 3B–D). In good correlation to our data, the differentiation of dendritic cells under hypoxia strongly reduced cytokine levels, e.g., IL-6, TNF-α, and IL-1β under low oxygen concentrations (defined as 1% O_2_), proving that hypoxia has a major influence on cytokine production (48). We suggest that the physioxically differentiated phenotype with less stored histamine, less ROS production in response to different stimuli and decreased gene expression of *hif-1*α and target genes, including important proinflammatory cytokines like *il-6* and *tnf-*α reflects the tissue status of MCs in *in vivo*. Since the differentiation of blood-derived monocytes into tissue macrophages under normoxia increases HIF and its target gene VEGF levels during the differentiation (63), atmospheric oxygen concentrations, namely normoxia, which are normally used to differentiate MCs *in vitro*, might reflect a stress situation for the cell due to an excessive oxygen level. Normoxia might, thus, resemble physiologically a hyperoxic state that provoke oxygen stress in MCs and explain their significant role in hyperoxia-induced lung injury (64). Oxygen stress may cause an upregulation of intracellular histamine levels, ROS production and various genes with all its consequences for immune cell recruitment, antimicrobial defense upon infection, and impact on tissue injury. However, the detailed consequences of MC differentiation under physioxia on MC functionality in response to infection and inflammation still needs to be investigated more in detail in future studies. Furthermore, it is still unclear if there might be differences to oxygen stress response in mucosal MCs versus connective tissue MCs.

## Conclusion

Our data show that physiological oxygen conditions substantially affect the maturation of MCs with regard to differentiation rate, gene expression of *hif-1*α and its target genes as well as *foxo3* and *vhl*, ROS production and cellular histamine storage. We could shed light on the relevance of experiments focusing on the key role of oxygen in cell culture. Finally, the differentiation under physiological conditions produces a different MC phenotype in comparison to normoxic differentiation, which more reflects the *in vivo* phenotype preferred for further application-oriented studies.

## Ethics Statement

This study was carried out in accordance with the recommendations of Nds. Landesamt für Verbraucherschutz und Lebensmittelsicherheit. The protocol was approved by the local ethical commission of Lower Saxony.

## Author Contributions

MK-B, KB-H, HM, and HN: conceived and designed the experiments; HM, KB-H, KS, KM, and HF: performed the experiments; HM, KM, MK-B, and KB-H: analyzed the data; HM, KB-H, and MK-B: wrote the paper. All authors proofread the paper.

## Conflict of Interest Statement

The authors declare that the research was conducted in the absence of any commercial or financial relationships that could be construed as a potential conflict of interest.

## Supplementary Material

Figure S1 Intracellular histamine storage after physioxic differentiation (Ctr) and stimulated with the mast cell (MC)-degranulating peptide mastoparan, measured by high performance liquid chromatography. The amount of intracellular stored histamine is significantly reduced in MCs differentiated under low oxygen levels. The histamine release from granules in response to mastoparan was still observable. Depicted are mean values and SEM from cells of *n* = 3 individual experiments (six mice, two mice pooled for each batch).
