# Supplementary figures and images for: Differentiation and Functionality of Bone Marrow-Derived Mast Cells Depend on Varying Physiologic Oxygen Conditions
Source: Front Immunol. 2017 Nov 30;8:1665. doi: 10.3389/fimmu.2017.01665 (PMC5714875; doi:10.3389/fimmu.2017.01665)

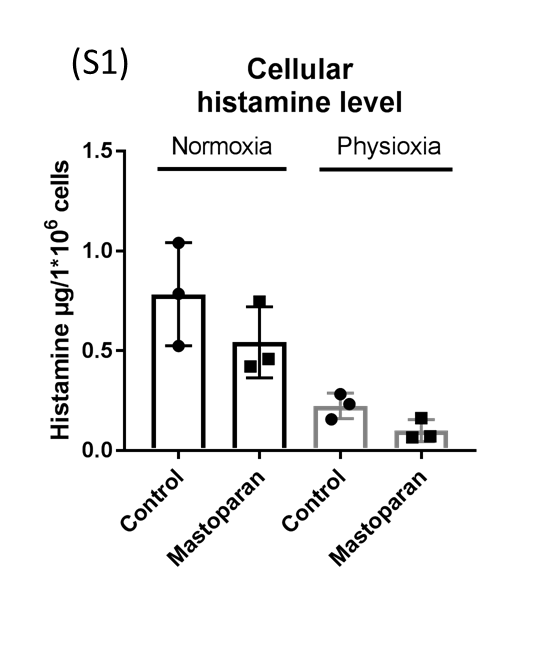

Supplement: Figure S1 — Intracellular histamine storage after physioxic differentiation (Ctr) and stimulated with the mast cell (MC)-degranulating peptide mastoparan, measured by high performance liquid chromatography. The amount of intracellular stored histamine is significantly reduced in MCs differentiated under low oxygen levels. The histamine release from granules in response to mastoparan was still observable. Depicted are mean values and SEM from cells of n = 3 individual experiments (six mice, two mice pooled for each batch). [file Image_1.tif]
